# Phylogenetic and Expression Studies of Small GTP-Binding Proteins in *Solanum lycopersicum* Super Strain B

**DOI:** 10.3390/plants11050641

**Published:** 2022-02-26

**Authors:** Hassan S. Al-Zahrani, Tarek A. A. Moussa, Hameed Alsamadany, Rehab M. Hafez, Michael P. Fuller

**Affiliations:** 1 Biological Sciences Department, Faculty of Science, King Abdulaziz University, Jeddah 21589, Saudi Arabia; hsalzahrani@kau.edu.sa (H.S.A.-Z.); halsamadani@kau.edu.sa (H.A.); 2 Botany and Microbiology Department, Faculty of Science, Cairo University, Giza 12613, Egypt; rehabhafez@sci.cu.edu.eg; 3 School of Biological and Marine Sciences, Faculty of Science and Engineering, University of Plymouth, Drake Circus, Plymouth PL4 8AA, UK; mfuller@plymouth.ac.uk

**Keywords:** *S. lycopersicum* super strain B, phylogenetic analyses, protein expression, protein sequence alignment, small GTPase superfamily

## Abstract

This investigation involved a comparative analysis of the small GTPase superfamily in *S. lycopersicum* super strain B compared to their analogues in leguminous and other non-leguminous species. The small GTPases superfamily members were recognized by tBLASTn searches. The sequences of amino acid were aligned using Clustal Omega and the analysis of phylogeny was performed with the MEGA7 package. Protein alignments were applied for all studied species. Three-dimensional models of RABA2, ROP9, and ROP10 from *Solanum lycopersicum* “Super strain B” were performed. The levels of mRNA of the Rab, Arf, Rop, and Ran subfamilies were detected in aerial tissues vs. roots. Significant divergences were found in the number of members and groups comprising each subfamily of the small GTPases and *Glycine max* had the highest count. High expression of Rab and Arf proteins was shown in the roots of legumes whilst in non-legume plants, the highest values were recorded in aerial tissues. *S. lycopersicum* super strain B had the highest expression of Rab and Arf proteins in its aerial tissues, which may indicate that diazotroph strains have supreme activities in the aerial tissues of strain B and act as associated N-fixing bacteria. The phylogenies of the small GTPase superfamily of the studied plants did not reveal asymmetric evolution of the Ra, Arf, Rop, and Ran subfamilies. Multiple sequence alignments derived from each of the Rab, Arf, and Rop proteins of *S. lycopersicum* super strain B showed a low frequency of substitutions in their domains. GTPases superfamily members have definite functions during infection, delivery, and maintenance of N_2_-fixing diazotroph but show some alterations in their function among *S. lycopersicum* super strain B, and other species.

## 1. Introduction

*Solanum lycopersicum* L. (tomato) is a vegetable crop cultivated all over the world for its high agro-economic importance [1]. It requires heavy manure and an adequate nitrogen supply to obtain the highest yields [2]. It appears that *S. lycopericum* obtains its nitrogen from both chemical fertilization with organic and inorganic manure [3] and acetylene reduction performed by diazotrophic bacteria present on the rhizoplane and the rhizosphere soil [4,5].

Various molecular components involved with diazotrophs infection have been highlighted as facilitating intracellular membrane trafficking [6,7,8], cytoskeleton related-proteins [9,10], and cell-wall degeneration enzymes. Among the proteins related to vesicle membrane trafficking are small GTPases, which have essential contributions to plant growth and development, including in the first diazotroph contagion process, root hair formation, and in the signaling pathway essential for nodulation in leguminous plants [7,8,11] and in the late symbiosome stage [12,13].

The small GTPases superfamily are commonly classified into five main subfamilies depending on their sequence, structure, and function identities: (1) Ras, (2) Rab, (3) Arf/Sar, (4) Rop/Roh, and (5) Ran [14,15], but only four of them are present in plants. Ras/Rab family members are key regulators that control the cell fate through specification, development, and differentiation [16], but Ras proteins are absent from plant genomes [15]. Arf/Sar are the main GTP-binding proteins that control morphogenesis, microtubule organization, and membrane trafficking joining the endoplasmic reticulum with the Golgi apparatus [16,17,18,19,20]. Individuals of the Rop family, noted as Rho of plants, are Ras homologous and regulate many processes in plants, such as polarized cell development, morphogenesis, cytoskeleton dynamics, hormone signaling, safeguard, and responses of the cell towards external stimuli [16,21,22]. However, Ran members (Ras-related nuclear protein) regulate nuclear import/export across the nuclear pore, mitotic nuclear reassembly, and kinetochore binding with microtubules [16,23].

Since these genes are crucial for cell vitality because of their housekeeping activities, they are well conserved functionally and sequentially among eukaryotes [24,25]. However, some alterations in their functional and expressional lineaments have been detected in some plants, indicating the possibility of acquiring linked or even new functions [15,26]. Therefore, transcriptome analyses are essential for collecting all sequence information from available plant species to investigate the degree of gene divergence between species-species and species-progenies [25,27].

The tomato cultivar super strain B is widely cultivated in Saudi Arabia and due to the nature of the soil in Saudi Arabia, which is almost desert, the plant can obtain its nutrients via chemical fertilization or via the degradable materials from microorganisms in the rhizosphere and rhizoplane of the plant. The GTPase family members are involved in nitrogen nutrition, so the present investigation aimed to detect the small GTPase family in the transcriptome of tomato cultivar “strain B” and compare the distinctive expression motif with their analogues in some leguminous and non-leguminous plants obtained from the database.

## 2. Results and Discussion

The count of each small GTPases subfamily member in *S. lycopersicum* “super strain B” compared to the non-leguminous and leguminous species is represented in Table 1. The table showed high divergence in the RAB number present in legume and non-legume species, where *G. max* recorded the highest member of RAB members (94). *L. japonicus* and *O. sativa* possessed the lowest number of members in the RAB subfamily. Between the highest and the lowest RAB number, the other species were 64 members in *M. truncatula*, followed by 57 in *A. thaliana*, 53 in *Z. mays*, 50 in *P. vulgaris*, and 46 in *S. lycopersicum* “super strain B”. Regarding the ARF subfamily, the maximum numbers were in *G. max* (41) and the least were in *L. japonicus* (13). The other species comprised nearly half the number of members in *G. max* (19–21), except in case of *Z. mays*, which has slightly higher numbers of members (23). Concerning the ROP subfamily, *G. max* still had the highest number of members (20) and the other legumes and non-legumes species had fewer. *P. vulgaris* and *A. thaliana* had 11 members, *S. lycopersicum* had 10, *Z. mays* and *S. lycopersicum* “super strain B” had 9, *L. japonicas* and *O. sativa* had 8, and finally, *M. truncatula* had the least members (7). Members of the RAN subfamily were higher in *G. max* (7) and lower in both *L. japonicus* and *O. sativa* (2). The other legumes and non-legume species possessed around three to four RAN members (Table 1). The results indicated the presence of significant variations in the score of members representing each subfamily of the small GTPases superfamily found in leguminous and non-leguminous species, in which *G. max* scored the highest number of members. According to Singh and Hymowitz [28], the drastic number of soybean GTPases subfamilies may refer to its genomic nature as a partially diploidized tetraploid species.

Table 2 illustrates the number of members in each group of the small GTPases Rab subfamily. Group A of the Rab subfamily had the highest number of members along with *S. lycopersicum* super strain B (186), in which the leguminous plants has more Rab participants (99) than the non-leguminous plants (87), despite the presence of the highest representatives in *G. max* (41). The other groups of the Rab subfamily acquired less than a quarter of the total members present in group A. From all groups, group B had the lowest total number of members (26), with nearly equal numbers of representatives in both legumes and non-legume species. Table 2 also reveals that the total number of members of Rab Group C was asymmetrically split between the legume and non-legume plants, in which the members in legumes (26) were about triple those in non-legumes species (9).

The number of members in each group of the small GTPases Arf subfamily is presented in Table 3. Group (B + C + D) had the highest number of members (58) among all studied species. Although the non-leguminous species had the same number of participants in the group (A + B + C), *G. max* still had the highest number of individuals (12). Group (ARLC) was the minor group of members, which consisted of a total of 9 members nearly equal distributed between leguminous and non-leguminous species. The majority of the Arf members in groups (A, ARLA, and ARLB) were from the non-leguminous plants as compared to the leguminous ones. However, the opposite was recorded in group SARA, where the highest total Arf numbers of individuals were detected in leguminous plants (19), which were mainly from *G. max* (10), Table 3.

Gene expression of the GTP protein families often appears to vary in spatio-temporal control between different species. Table 4 delineated the amounts of mRNA expressed in the members of each small GTPases subfamily of *S*. *lycopersicum* super strain B, and other non-legume and legume plants, present in aerial tissue vs. those of root. Using root samples as a reference, normalized values were derived for cross referencing to other tissues. In all species in this study, GTPases were almost accumulated at higher levels in the roots compared to aerial tissues. The members of the Rab and Rop subfamilies of *S*. *lycopersicum* super strain B showed a higher mRNA level in their aerial tissues compared to the roots, consistent with those in the other species, except in P. vulgaris and G. max, where Rops also demonstrated increased levels of mRNA in its aerial tissues (Table 4). Only one member of Ran subfamily in both *O. sativa* and *L. japonicus* had high mRNA while the other species had none. All species have members of the Rab, Arf, and Rop subfamilies with consistent amounts of mRNA levels in their aerial tissues, in which *G. max* had higher levels (Table 4). The results of analyzing the expression of small GTPases subfamily members in both leguminous and non-leguminous species indicated that a higher number of Rab and Arf members were upregulated in aerial tissues than roots in non-leguminous plants, especially in *S*. *lycopersicum* super strain B. However, the members of each subfamily (Rab, Arf, Rop, and Ran) with unchanged levels of mRNA in aerial tissues were comparable in leguminous and non-leguminous species of the study. In addition, the highest downregulation of each small GTPases subfamily member was observed in *G. max*. The high accumulation of mRNA of both Rab and Arf proteins in the roots of legumes may indicate that they are the main proteins involved in the symbiotic relation between legumes and rhizobia. Probable tissue-specific functionalization of Rab/Arf small-GTP binding genes/proteins was suggested to participate in the genesis, development, and maintenance of nodulations in the roots of legume plant as reported by several investigators of Rab in soybean and *Vigna* *aconitifolia* [29], *Lotus* *japonicus* [30], soybean [31], *Medicago sp* [12,32], kidney bean [6], and Rab/Arf in *Medicago truncatula* [13]. Concerning non-leguminous plants, the high expression of Rab and Arf proteins in the aerial tissues, especially in *S. lycopersicon* strain B, may disclose the presence of another mechanism different from nodulations that involve N_2_ uptake and fixation. In this context, Mohandas [33] revealed the domestication of some rhizobacteria in the roots and leaves and on the rhizoplane and phylloplane of tomato (*L. esculentum* Mill “Pusa Ruby”) as associated N_2_-fixing bacteria. In addition, Dent and Cocking [34] reported that diazotroph strains can intracellularly colonize, under specific conditions, the roots (or root hairs) and shoots of non-legume plants without nodulation in cereals, such as wheat, maize, and rice, in addition to some crops, such as potato, oilseed rape, and tomato. Moreover, Collavino et al. [35] reported that the diazotrophic populations inside the stem and root of tomato plants play a critical function in the early growth phases and are distinctively influenced by N fertilization. From all the above, we can speculate that the high gene expressions may mean that diazotroph strains are colonized more in the aerial tissues than the roots (either inside or on the surface) of *S. lycopersicum* super strain B.

**Figure 1:**
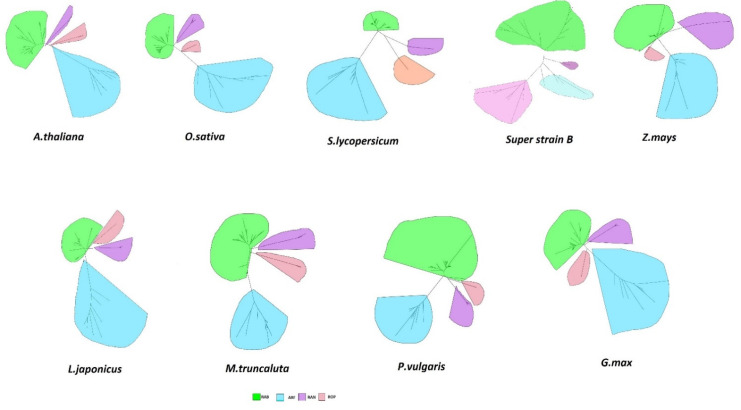
Phylogenetic analysis of the small GTPase superfamily in *S. lycopersicum super strain B* and other legume and non-legume plants. Amino acid sequences corresponding to small GTPases from *O. sativa*, *A. thaliana*, *S. lycopersicum*, *L. japonicus*, *Z. mays*, *M. truncatula*, *G. max*, and *P. vulgaris* were restored from genomic databases. Unrooted neighbor-joining trees were obtained using the Mega 7 software. Subfamilies were recognized for each species: Rab (green), Arf (blue), Rop (Pink), and Ran (violet).

Monomeric GTPase sequences of amino acids from tBLASTn searches were applied to recognize the individuals of the small GTPases superfamily of *S. lycopersicum* super strain B and those retrieved from the genomic databases (*O. sativa*, *A. thaliana*, *S. lycopersicum*, *L. japonicus*, *Z. mays*, *M. truncatula*, *G. max*, and *P. vulgaris*). Amino acid sequences of those proteins were employed to create phylogenetic trees of those species, permitting their categorization using small GTPases subfamilies into Rab (green), Arf (blue), Rop (Pink), and Ran (violet), (Figure 1). The phylogenetic inspection of the small GTPase superfamily of the studied leguminous and non-leguminous plants did not reveal asymmetric evolution of the Ra, Arf, Rop, and Ran subfamilies. These results were in accordance with those of Flores et al. [27].

**Figure 2:**
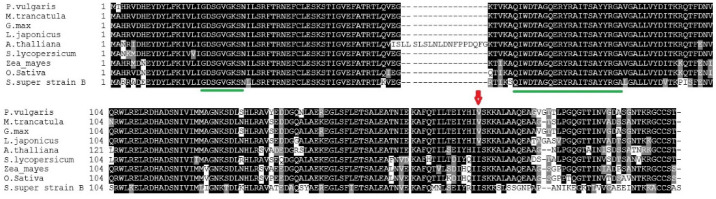
Multiple sequence alignments and the proteins of *S. lycopersicum super strain B* (RABA2) and those retrieved from the genomic databases: *A. thaliana* (At1g07410, *O. sativa* (LOC_Os03g60870), *S. lycopersicum* (Solyc06g076450), *Z. mays* (GRMZM2G473906), *L. japonicus* (chr3.CM0792.300.r2.d), *M. truncatula* (Medtr4g064897), *P. vulgaris* (Phvul.011G061100), and *G. max* (Glyma11g14360). Black boxes represent the identical residues while gray ones represent conservative substitutions. Alignments were performed with Clustal Omega in MEGA7 followed with Boxshade. The red arrow designates a conservative amino acid substitution in legume against non-legume sequences. The conserved domains of Rabs are indicated by green lines.

Multiple sequence alignments of RABs proteins of *S. lycopersicum* super strain B, *O. sativa*, *A. thaliana*, *S. lycopersicum*, *L. japonicus*, *Z. mays*, *M. truncatula*, *G. max*, and *P. vulgaris* are illustrated in Figure 2. *S. lycopersicum* super strain B showed 2 RABs strong amino acid conserved domains, which was similar to those of the other legume and non-legume species. RABA2 proteins showed conserved substitutions in the position 177 (out of domains), whereas a valine residue (V) was found in 4 leguminous species (*P. vulgaris* RABA2, *M. truncatula*, *L. japonicus*,and *G. max*) and a isoleucine (I) in *S. lycopersicum* super strain B and the other non-legume species (*A. thaliana*, *O. sativa*, *S. lycopersicum*, *Z. mays*) (Figure 2).

**Figure 3:**
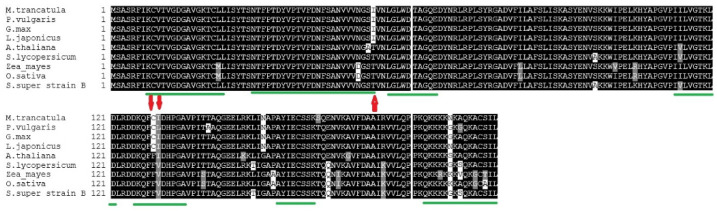
Multiple sequence alignments and the proteins of *S. lycopersicum super strain B* (ROP9) and those retrieved from the genomic databases: *A. thaliana* (At2g17800), *O. sativa* (LOC_Os02g58730), *S. lycopersicum* (Solyc02g083580), *Z. mays* (GRMZM2G375002), *L. japonicus* (chr2.CM0272.860.r2.m), *M. truncatula* (Medtr4g064897), *P. vulgaris* (Phvul.002G106600), and *G. max* (Glyma01g36880). Black boxes represent the identical residues while gray ones represent conservative substitutions. Alignments were performed with Clustal Omega in MEGA7 followed with Boxshade. The red arrow designates a conservative amino acid substitution in legume against non-legume sequences. The conserved domains of Rabs were indicated by green lines. The conserved domains of ROPs were indicated by green lines.

All alignments of ROP9 proteins manifested powerful amino acid sequence conservation across the studied plants, which were clarified by the presence of 7 domains (Figure 3). Three positions (amino acids 53, 129, and 130) showed conserved substitutions in the domains of ROP9 proteins, and 3 other substitutions were out of it (amino acids 151, 164, 175). The former substitution was at the border of II, where isoleucine in leguminous species was switched with threonine (T) in non-leguminous ones. The second and the third ones were in the mid-region of the domain number V. In the second substitution, cysteine (C) was found in leguminous plants and phenylalanine (F) in non-leguminous ones. Interestingly, the third substitution was variable, in which a valine residue was recorded in only 4 non-leguminous plants (*O. sativa*, *S. lycopersicum*, *Z. mays*, *S. lycopersicum* super strain B). *A. thaliana*, however, differed from its non-legume species and has isoleucine residue like the leguminous ones except for *P. vulgaris*, which has leucine (L) instead (Figure 3).

**Figure 4:**
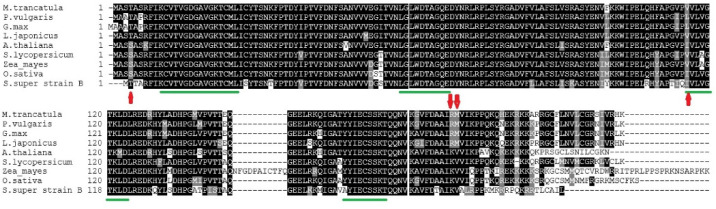
Multiple sequence alignments and the proteins of *S. lycopersicum super strain B* (ROP10) and those retrieved from the genomic databases: *A. thaliana* (At1g07410, *O. sativa* (LOC_Os03g60870), *S. lycopersicum* (Solyc06g076450), *Z. mays* (GRMZM2G473906), *L. japonicus* (chr3.CM0792.300.r2.d), *M. truncatula* (Medtr4g064897), *P. vulgaris* (Phvul.011G061100), and *G. max* (Glyma11g14360). Black boxes represent identical residues while gray ones represent conservative substitutions. Alignments were performed with Clustal Omega in MEGA7 followed with Boxshade. Red arrows designate amino acid substitutions in legumes against non-legumes. The conserved domains of ROP are indicated by green lines.

By aligning the sequence of ROP10 proteins of all the species studied, we found the presence of 4 domains. ROP10 protein alignments also possessed substitutions in legume species (Figure 4), but only one of those swaps affects the ROP domain. This substitution was at the beginning of III, where leucine was found in *P. vulgaris* and *G. max*, valine in *M. truncatula*, *L. japonicas*, *S. lycopersicum* and *Z. mays*, and isoleucine in *A. thaliana*, *O. sativa* and *S. lycopersicum* super strain *B.*

**Figure 5:**
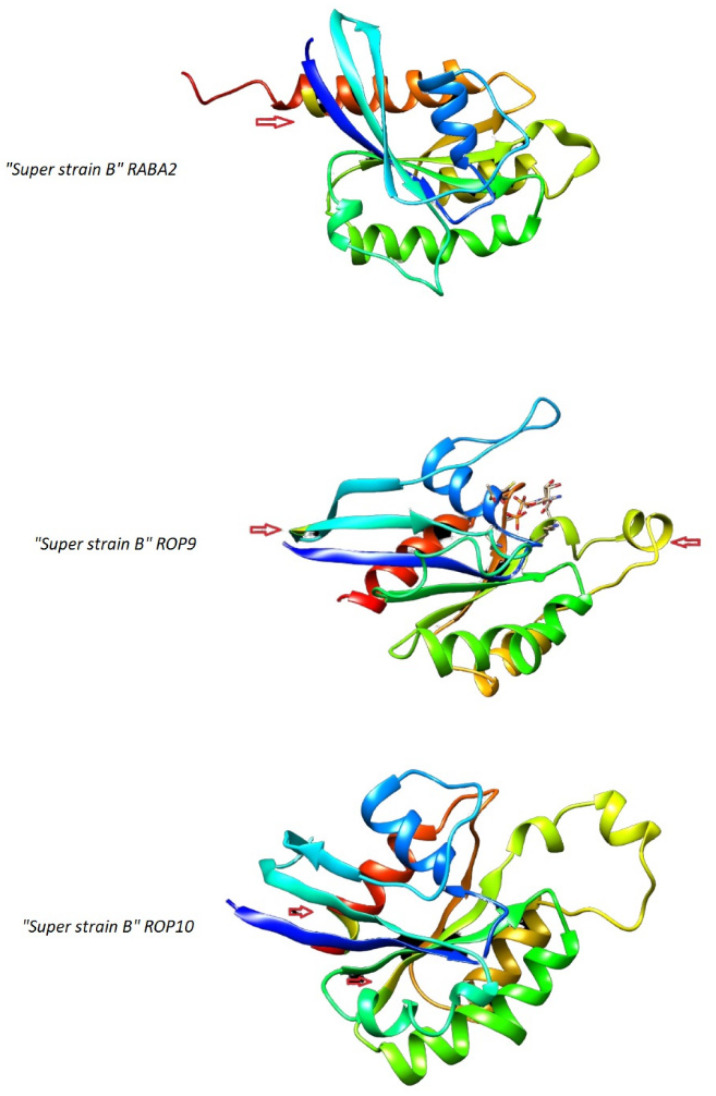
Three-dimensional models of RABA2, ROP9, and ROP10 from *Solanum lycopersicum* “Super strain B”. Arrows and yellow boxes indicate the position of the substitutions noticed in super strain B versus other legume and non-legume species.

By comparing the sequence alignment of the RABA2, ROP9, and ROP10 proteins of *S. lycopersicum* super strain B to their analogues in non-legume and legume plants, we found that RABA2 has a single conserved substitution while ROP9 and ROP10 have three (Figure 2, Figure 3 and Figure 4). Those substitutions were affirmed by the predicted 3D configurations of RABA2, ROP9, and ROP10 proteins of *S. lycopersicum super strain B* (Figure 5). Multiple sequence alignments that were obtained for each of Rab, Arf, and Rop proteins of *S. lycopersicum super strain* B and their analogues in non-legume and legume plants as illustrated in Figure 2, Figure 3 and Figure 4 showed a low frequency of substitutions in their domains. This may indicate the strong conservation of amino acid sequence across the leguminous and non-leguminous plants analyzed and it is proposed that those proteins were put through powerful discriminatory pressure as reported by Flores et al. [27]. Multiple sequence alignments of the leguminous plants’ proteins revealed that the domains of RABs proteins had no conserved substitutions while both ROP9 and ROP10 had one each. ROP9 showed a conserved substitution (in amino acid 130) in domain V where leucine was in *P. vulgaris* (Phvul.002G106600) and isoleucine was in the analogue of the other three legumes [*L. japonicus* (chr2.CM0272.860.r2.m), *M. truncatula* (Medtr4g064897), and *G. max* (Glyma01g36880)]. Moreover, ROP10 had conserved residues (in amino acid 115) in domain III where leucine was found in kidney bean (Phvul.009G180800) and soybean (Glyma04g35110) while valine in *M. truncatula* ROP10 (Medtr3g078260) and *L. japonicus* (chr1.CM0166.830.r2.m). Jiang and Ramachandran [24] and Yuksel and Memon [36] reported that small GTP-binding proteins in most plants are functionally very well conserved, but some could follow functional variations in divergent lineages to regulate some lineage-specific functional roles, such as nodulation in legume plants. So, the variations in the conserved residues in those legume plants may reflect the specific contribution of those proteins in the legumes–rhizobia symbiosis relationship [27].

## 3. Materials and Methods

### 3.1. Identification of Small GTPases from Different Species

The small GTPases superfamily members were recognized by tBLASTn searches [37] using the amino acid sequence of all small GTPase family individuals that were previously described and categorized in *Arabidopsis* [15,26]. These genes were selected and categorized manually following a systematic phylogenetic analysis.

### 3.2. Phylogenetic Analysis

The sequences of amino acids were aligned using Clustal Omega (http://www.ebi.ac.uk/Tools/msa/clustalo (accessed on 15 March 2020)) [38] and the analysis of phylogeny was carried out with the MEGA7 package (http://www.megasoftware.net (accessed on 15 March 2020)) [39] using the method of neighbor-joining [40]. The distances of evolution were computed by the difference’s method [41]. All positions of gaps and data missing were omitted from the dataset.

### 3.3. Protein Alignments

Small GTPases amino acid sequences that participated in the initiation of the symbiotic relation between rhizobia and legumes were applied to identify members from other species by BLASTP. Te PvRabA2 and PvArfA1 were selected from *Phaseolus vulgaris* (common bean), LjRop6 from *Lotus japonicus*, and MtRab7, MtRop9, and MtRop10 from *Medicago truncatula* as queries. The sequences with the lowest E value were applied to create multiple alignments by Clustal Omega (http://www.ebi.ac.uk/Tools/msa/clustalo accessed on 15 March 2020)) and organized with Boxshade (http://embnet.vital-it.ch/software/BOX_form.html (accessed on 15 March 2020)).

### 3.4. D Modeling

Swiss Model [42] (https://swissmodel.expasy.org/ (accessed on 15 March 2020)) was used for protein modeling and the 3D structure viewer iCn3D (https://www.ncbi.nlm.nih.gov/Structure/icn3d/full.html (accessed on 15 March 2020)) for analysis.

### 3.5. Genomic Datasets

Sequences of *Arabidopsis thaliana* (TAIR10), *Glycine max* (Wm82.a2.v1), *Medicago truncatula* (Mt4.0v1), *Oryza sativa* (v7_JGI), *Solanum lycopersicum* (iTAG2.3), and *Zea mays* (Ensembl-18) were obtained from datasets. *Lotus japonicus* (v2.5) was obtained from Miyakogusa v2.5 (http://www.kazusa.or.jp/lotus (accessed on 15 March 2020)) and *Phaseolus vulgaris* (v1.0) from Phytozome v11.0 (https://phytozome.jgi.doe.gov/pz/portal.html (accessed on 15 March 2020)).

### 3.6. Transcriptomic Datasets

Small GTPases gene expression of *A. thaliana*: expression data were obtained from TraVA (http://travadb.org (accessed on 15 March 2020)) [43], *S. lycopersicum* cv. Heinz (http://ted.bti.cornell.edu (accessed on 15 March 2020)) (TGC 2012), *O. sativa* cv. Nippon bare [44], *Z. mays* from RNAseq transcriptomic analyses [45], *M. truncatula* cv Jemalong A17 from MtGEA (http://mtgea.noble.org/v3 (accessed on 15 March 2020)) [46], *L. japonicus* from LjGEA (http://ljgea.noble.org/v2 (accessed on 15 March 2020)) [47], *P. vulgaris* cv. NAG12 from (http://plantgrn.noble.org/PvGEA (accessed on 15 March 2020)) [48], and *G. max* from SoyBase (https://www.soybase.org/soyseq (accessed on 15 March 2020)) [49] was retrieved using databases available in the public domain.

### 3.7. Statistical Analysis of Expression Data

Small GTPase members were retrieved using the public datasets for each species. Their expressions manifested in roots (reference organ) and in other organs of the eight species in study. The values of expression were normalized for each tissue and the statistical analyses were achieved using CuffDiff [50] to identify the expressed genes.

## 4. Conclusions

The highest numbers of Rab and Arf proteins were expressed in tomato super strain B and all the species compared in this study. The levels of Rab and Rop mRNA in aerial tissues were higher than in roots, but in contrast, Arf mRNA levels was higher in roots than in aerial tissues. The Ran subfamily showed the least expression in different tissues of tomato super strain B.

## Figures and Tables

**Table 1:** Number of each small GTPases subfamily members expressed in *Solanum lycopersicum* super strain “B” compared with some non-leguminous and leguminous plants retrieved from the data base.

GTPases	*S. lycopersicum* Super Strain “B”	Non-Legumes			Legumes			
		*A. thaliana*	*O. sativa*	*Z. mays*	*L. japonicus*	*M. truncatula*	*P. vulgaris*	*G. max*
RAB	46	57	37	53	30	64	50	94
ARF	21	21	21	25	13	19	20	41
ROP	9	11	8	9	8	7	11	20
RAN	4	4	2	3	2	4	3	7
Total	80	93	68	90	53	94	84	162

**Table 2:** Number of the small GTPases Rab subfamily members expressed in *Solanum lycopersicum* super strain “B” compared with some non-leguminous and leguminous plants retrieved from the data base.

Group	*S. lycopersicum* Super Strain “B”	Non-Legumes			Legumes			
		*A. thaliana*	*O. sativa*	*Z. mays*	*L. japonicus*	*M. truncatula*	*P. vulgaris*	*G. max*
A	21	26	17	23	12	23	23	41
B	2	3	3	4	2	7	1	4
C	3	3	0	3	4	6	5	11
D	5	4	4	6	1	4	4	7
E	5	5	3	5	3	6	5	8
F	4	3	4	3	3	5	4	7
G	3	8	4	5	3	9	4	8
H	3	5	2	4	2	4	4	8
Total	46	57	37	53	30	64	50	94

**Table 3:** Number of small GTPases Arf subfamily members expressed in *Solanum lycopersicum* super strain “B” compared with some non-leguminous and leguminous plants retrieved from the data base.

Group	*S. lycopersicum* Super Strain “B”	Non-Legumes			Legumes			
		*A. thaliana*	*O. sativa*	*Z. mays*	*L. japonicus*	*M. truncatula*	*P. vulgaris*	*G. max*
A	5	6	6	6	4	5	4	10
B + C + D	8	6	6	9	4	7	6	12
ARLA	3	4	2	4	1	3	4	5
ARLB	1	1	2	2	1	0	1	2
ARLC	1	1	1	1	1	1	1	2
SARA	3	3	4	3	2	3	4	10
Total	21	21	21	25	13	19	20	41

**Table 4:** The number of each GTPase subfamily member that shows no change (N), reduced (−), or increased (+) levels of mRNA in *Solanum lycopersicum* super strain “B” compared with some non-leguminous and leguminous plants retrieved from the database in aerial tissue vs. the root.

Plant Species	Rab			Arf			Rop			Ran		
	−	N	+	−	N	+	−	N	+	−	N	+
*S. lycopersicum* super strain “B” ^a^	8	24	14	8	8	5	1	3	5	0	4	0
*A. thaliana* ^a^	16	36	5	6	13	2	4	7	0	0	4	0
*O. sativa* ^b^	14	20	2	4	16	1	2	4	2	0	1	1
*Z. mays* ^c^	17	34	0	5	18	1	0	8	1	0	3	0
*L. japonicus* ^d^	1	29	0	0	13	0	2	6	0	0	1	1
*M. trancatula* ^a^	17	26	2	5	13	0	2	5	0	2	2	0
*P. vulgaris* ^a^	14	28	3	5	13	0	1	6	4	1	2	0
*G. max* ^a^	50	42	2	19	20	2	5	9	6	4	3	0

Note: ^a^ compared to leaf; ^b^ compared to shoot (2-week-old); ^c^ compared to stem; ^d^ compared to leaf (6-week-old).

## Data Availability

The data presented in this study are available in insert article or Supplementary Material here.

## Supplementary Materials

The following are available online at https://www.mdpi.com/article/10.3390/plants11050641/s1, Table S1: Expression data of the *Arabidopsis thaliana* small GTPase superfamily, Table S2: Expression data of the *Oryza sativa* small GTPase superfamily by RNA sequencing, Table S3: Expression data of the *Solanum lycopersicum* small GTPase superfamily, Table S4: Expression data of the *Zea mays* small GTPase superfamily by RNA sequencing, Table S5: Expression data of the *Lotus japonicus* small GTPase superfamily, Table S6: Expression data of the *Medicago truncatula* small GTPase superfamily by microarray, Table S7: Expression data of the Phaseolus vulgaris small GTPase superfamily by RNA sequencing, Table S8: Expression data of the Glycine max small GTPase superfamily by RNA sequencing, Table S9: RNA-seq data for M. truncatula small GTPases detected in different regions of the nodule.
